# Determination of Integral Depth Dose in Proton Pencil Beam Using Plane-parallel Ionization Chambers

**DOI:** 10.14338/IJPT-22-00006.1

**Published:** 2022-06-03

**Authors:** Phatthraporn Thasasi, Sirinya Ruangchan, Puntiwa Oonsiri, Sornjarod Oonsiri

**Affiliations:** 1 Medical Physics Program, Faculty of Medicine, Chulalongkorn University, Bangkok, Thailand; 2 Division of Radiation Oncology, Department of Radiology, King Chulalongkorn Memorial Hospital, Bangkok, Thailand

**Keywords:** proton therapy, pencil beam scanning, integral depth dose

## Abstract

**Purpose:**

This study aimed to determine the integral depth-dose curves and assess the geometric collection efficiency of different detector diameters in proton pencil beam scanning.

**Materials and Methods:**

The Varian ProBeam Compact spot scanning system was used for this study. The integral depth-dose curves with a proton energy range of 130 to 220 MeV were acquired with 2 types of Bragg peak chambers: 34070 with 8-cm diameter and 34089 with 15-cm diameter (PTW), multi-layer ionization chamber with 12-cm diameter (Giraffe, IBA Dosimetry), and PeakFinder with 8-cm diameter (PTW). To assess geometric collection efficiency, the integral depth-dose curves of 8- and 12-cm chamber diameters were compared to a 15-cm chamber diameter as the largest detector.

**Results:**

At intermediate depths of 130, 150, 190, and 220 MeV, PTW Bragg peak chamber type 34089 provided the highest integral depth-dose curves followed by IBA Giraffe, PTW Bragg peak chamber type 34070, and PTW PeakFinder. Moreover, PTW Bragg peak chamber type 34089 had increased geometric collection efficiency up to 3.8%, 6.1%, and 3.1% when compared to PTW Bragg peak chamber type 34070, PTW PeakFinder, and IBA Giraffe, respectively.

**Conclusion:**

A larger plane-parallel ionization chamber could increase the geometric collection efficiency of the detector, especially at intermediate depths and high-energy proton beams.

## Introduction

In the last decade, proton therapy with pencil beam scanning techniques, implemented in many proton facilities, gained more importance in radiation oncology because it provides dose escalation and more target dose conformity than other treatment techniques. These properties provide superior sparing of normal tissues and reduce radiation side effects and secondary cancer [1]. Proton spot characteristics have to be considered in dose calculation algorithms for clinical treatment planning systems, directly impacting the treatment quality. The input data needed for the proton dose configuration in the treatment planning systems are the integral depth-dose curves, absolute dose calibration, and spot profiles in the air [2]. This study focuses on the integral depth-dose curve because it is an essential factor that has to be determined and introduced into the proton treatment planning system before clinical use and it also represents beam quality and physical characteristics of the proton beam.

The integral depth-dose curve is the total dose on an infinite plane normal to the beam's central axis along the depths [3]. The proton pencil beam's initial size is a few millimeters, but the beam gets broadened owing to secondary protons and multiple scattering. The halo is one of the broad beam's components. It is the deposited dose around the primary beam and is mainly produced by large scattered secondary protons. It results from nuclear and coulomb interactions of the primary proton beam in the nozzle and detection medium (eg, water). The halo radius is approximately one-third of the beam range and the contribution is most pronounced at intermediate depths and the highest energy proton beam [4–7]. It could be extended up to a 10-cm radius, resulting in a dose increase of up to 15% due to overlapping halos from radial contributions of many neighboring pencils in a scanned beam [3].

Static and monoenergetic pencil beams should be delivered for the integral depth-dose curve measurements. Owing to the halo, there is a missing dose deposited outside the active area of the chamber. Therefore, accurate measurement of integral depth-dose curves for the treatment planning systems' beam configuration is required to reduce the inaccuracy of the halo effect. The report of AAPM (American Association of Physicists in Medicine) Task Group 185 [3] recommends that the integral depth-dose curve should be measured with a large-diameter plane-parallel ionization chamber to acquire the entire pencil beam and to improve the geometric collection efficiency, as evaluated by comparing integral depth-dose curves from 2 different chambers. It is efficient for a detector to collect the missing dose that associates with halo, nonelastic hadronic collision, and light fragments that are not measured by the chamber [8]. The most frequently used in clinical proton centers is PTW (Freiburg, Germany) Bragg peak chamber type 34070 with an 8-cm diameter. However, Langner et al [2] and Baumer et al [4] have indicated that for higher energies, PTW Bragg peak chamber type 34070 is not large enough to capture all secondary particles from the proton pencil beam, and Saini et al [9] found that the differences between measured and calculated doses increase with larger field sizes and higher energy, causing the treatment planning system to underestimate doses. Moreover, Baumer et al [4] found that the IBA Stingray chamber (IBA Dosimetry, Schwarzenbruck, Germany) with a 12-cm diameter requires 2% and 3.5% less correction than the PTW Bragg peak chamber type 34070 for 180 and 226.7 MeV, respectively.

Currently, there are many commercially available large-diameter ionization chambers, such as PTW PeakFinder with 8-cm diameter, IBA Stingray chamber with 12-cm diameter, IBA Giraffe with 12-cm diameter, and PTW Bragg peak chamber type 34089 with 15-cm diameter. Consequently, this study aims to determine the integral depth-dose curves and assess the geometric collection efficiency of 4 different detectors with diameters of 8, 12, and 15 cm in proton pencil beam scanning.

## Materials and Methods

### Varian ProBeam Compact System

The Varian ProBeam Compact spot scanning system (Varian Medical System, Palo Alto, California) uses a proton pencil beam with variable intensity and energy that magnetically steers across the target. The diameter and range of the pencil beam vary depending on energy. The energy selection system was used to degrade the beam and produced various lower energies. The lowest proton energy is 70 MeV, corresponding to 4.0 cm in water, and 220 MeV for 30.5 cm in water as the highest proton energy, allowing for treatment of the shallower depths. Proton spot sizes are between 3.5 and 6.0 mm (sigma), increasing diameter for lower energies. After leaving the energy selection system, the proton beam travels in a vacuum within the beamline. It is guided by various magnets, including dipole and quadrupole magnets, which can deflect and focus the beam. The gantry can be rotated 360° around a patient [10].

### Detectors

#### PTW Bragg peak chamber type 34070 (BP8)

The electrode diameter is 8 cm with an electrode spacing of 2 mm. The sensitive volume is 10.5 cm^3^. According to the vendor, the entrance window consists of 3.35-mm polymethyl methacrylate, corresponding to a water-equivalent thickness (WET) of 4.0 mm. The chamber is mounted in an MP3-PL water phantom (PTW) to acquire the integral depth-dose curves.

#### PTW Bragg peak chamber type 34089 (BP15)

The PTW Bragg peak chamber type 34089 is a large-area ionization chamber with a 15-cm electrode diameter with an electrode spacing of 2 mm. The sensitive volume is 34 cm^3^. According to the vendor, the entrance window consists of 2.47-mm carbon fiber–reinforced polymer, corresponding to a WET of 4.65 mm. This chamber is mounted in an MP3-PL water phantom, the same as PTW Bragg peak chamber type 34070.

#### PTW PeakFinder

The PTW PeakFinder is a closed water column containing approximately 6 L of distilled water with anticorrosion fluid and designed especially for the highest precision peak detection with a spatial resolution of 10 μm. The measuring chamber is a thin-window Bragg peak chamber type 34080, the same as thick-window Bragg peak chamber type 34070 with an 8-cm diameter. The signals are read out by the TANDEM XDR electrometer. The PTW PeakFinder does not have an absolute depth calibration; therefore, it has to be calibrated once before use by adjusting the offset parameter in PeakScan. In this study, the water equivalent depth offset is 13.35 mm.

#### IBA Giraffe

The Giraffe (IBA Dosimetry) is a large electrode multi-layer ionization chamber designed to measure the longitudinal depth-dose distribution of central-axis proton pencil beams and is composed of 180 plane-parallel ionization chambers fabricated with printed circuit board technologies. The outer graphite layers of each printed circuit board plate form the circular electrodes with a diameter of 12 cm and a detector spacing of 2 mm. The air gap between the 2 plates is approximately 1 mm. The WET of each channel is set to a value between 1.85 mm and 1.90 mm by the vendor. The effective points of measurements range from about 2 mm to 330 mm in a depth axis. A uniformity calibration should be performed before the operation of the multi-layer ionization chamber to correct the relative dose of each channel to match the reference measurement in water [4, 11]. The IBA Giraffe was calibrated in this study by using 220 MeV for PTW Bragg peak chamber type 34070, measured in a water phantom.

#### PTW x-ray therapy monitor chamber type 7862

It is used as a reference chamber with a diameter of 9.65 cm and a physical window thickness of 0.2 mm [2]. The WET of the chamber is 0.3 mm. The reference chamber was placed between the gantry and the measuring chamber at the center of the beam.

### Characteristics of the Detectors

Before measuring the integral depth-dose curves, all chambers were tested for short-term reproducibility, linearity, and repetition rate dependence. The study of these characteristics was undertaken with 150-MeV proton beams.

#### Short-term reproducibility

The IBA Giraffe was irradiated for 100 MU and the other chambers were irradiated for 10 000 MU repeatedly 10 times.

#### Linearity

The linearity was measured for MU settings ranging from 10 to 1000 MU and 500 to 50 000 MU with IBA Giraffe and the other chambers.

#### Repetition rate dependence

Fixed 100 and 10 000 MU were delivered to IBA Giraffe and to the other chambers, respectively, with different dose rates (50 000; 100 000; 750 000; 1 500 000; 3 000 000 MU/min). Values were normalized to 750 000 MU/min for all chambers.

### Measurement of the Integral Depth-Dose Curves

Static and monoenergetic pencil beams with proton energies of 130, 150, 190, and 220 MeV were delivered to measure the integral depth-dose curves, using 2 types of PTW Bragg peak chamber, 34070 and 34089, mounted in an MP3-PL water phantom for vertical beamline at gantry angle 0°, and PTW PeakFinder and IBA Giraffe for horizontal beamline at gantry angle 90°. The isocenter was placed on the water surface at the beam's central axis or on the PTW PeakFinder and IBA Giraffe marks.

For PTW Bragg peak chamber and PTW PeakFinder, we divided the step size into 3 parts: 5 mm for 130 and 150 MeV and 10 mm for 190 and 220 MeV (the plateau region), 0.5 mm (the peak region), and 5 mm (the distal fall-off region). The measured integral depth-dose curves were corrected for the WETs of the reference chamber and the entrance window of the chamber.

### Comparison of Bragg Peak Range

Bragg peak range (R_80_) was compared to ranges from the National Institute of Standards and Technology (NIST) [12], obtained by assuming continuous slowing down approximation without considering multiple coulomb scattering and nuclear interaction to validate setup positions, beam energies, and WETs of the entrance window of the chambers according to the vendor.

### Comparison of the Integral Depth-Dose Curves

All integral depth-dose curves were scaled to a maximum of 100% of the dose and shifted in depth to match R_80_ from NIST to eliminate the range uncertainty before comparing the integral depth-dose curves measured with PTW Bragg peak chamber type 34070, PTW Bragg peak chamber type 34089, PTW PeakFinder, and IBA Giraffe [4, 13].

### Assessment of the Geometric Collection Efficiency

The geometric collection efficiency of detectors was calculated at half of R_80_ (the intermediate depth) by comparing PTW Bragg peak chamber type 34070, PTW PeakFinder, and IBA Giraffe to PTW Bragg peak chamber type 34089, as shown in the following equation where IDD represents the integral depth-dose curve, d represents the chamber diameter of 8 and 12 cm, and z represents depth.



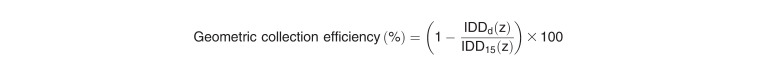




## Results

### Characteristics of the Detectors

#### Short-term reproducibility

The coefficient of variation was less than 0.2% for all chambers, with a minimum of 0.04% for 2 types of PTW Bragg peak chamber, indicating good short-term reproducibility.

#### Linearity

In terms of MU linearity, the response of all chambers was linear to the MU setting. Values of *R*^2^ > 0.99 with the linear function were found for all chambers.

#### Repetition rate dependence

Repetition rate dependence was within 0.3% for all chambers.

### Bragg Peak Range

The R_80_ differences between NIST and the measured values of all chambers from 130 to 220 MeV are shown in the **Table**. R_80_ differences were found to be within a 1-mm tolerance [14], with a maximum of 0.9 mm for IBA Giraffe at 190 MeV. This indicates the accuracy of setup positions of all chambers, beam energies, and WETs of the entrance window according to the vendor.

**Table.** Bragg peak range comparison between NIST and measured values of 130, 150, 190, and 220 MeV.

**Energy, MeV**	**NIST, mm**	**R_80_ measured, mm**				**R_80_ difference, mm**			
		**BP8**	**BP15**	**PeakFinder**	**Giraffe**	**BP8**	**BP15**	**PeakFinder**	**Giraffe**
130	122.6	122.4	122.6	122.4	122.2	−0.2	0.0	−0.2	−0.4
150	157.6	157.3	157.6	157.4	157.1	−0.3	0.0	−0.2	−0.5
190	237.4	237.3	237.5	237.2	236.5	−0.1	0.1	−0.2	−0.9
220	305.2	304.9	305.1	304.8	305.2	−0.3	−0.1	−0.4	0.0

**Abbreviations:** NIST, National Institute of Standards and Technology; BP8, Bragg peak chamber type 34070; BP15, Bragg peak chamber type 34089.

### The Integral Depth-Dose Curves

**Figure 1.**
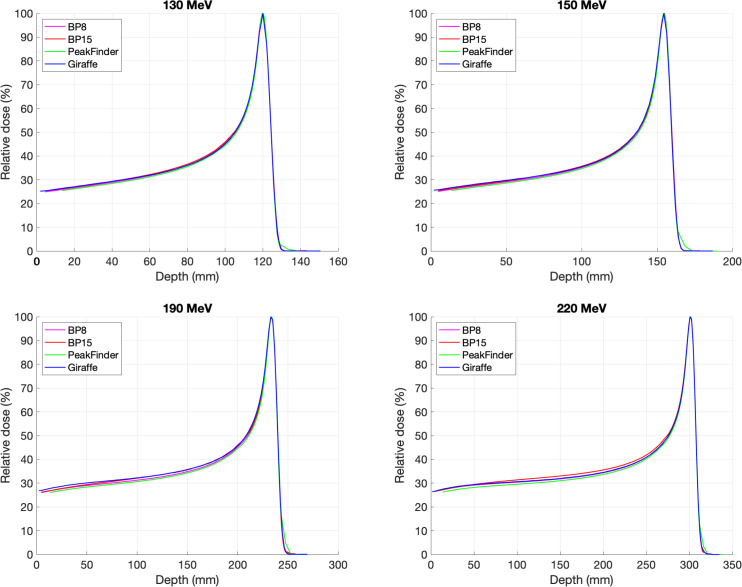
The integral depth-dose curves acquired with PTW Bragg peak chamber type 34070 (pink line), PTW Bragg peak chamber type 34089 (red line), PTW PeakFinder (green line), and IBA Giraffe (blue line) for 130, 150, 190, and 220 MeV. Abbreviations: BP8, Bragg peak chamber type 34070; BP15, Bragg peak chamber type 34089.

**Figure 2.**
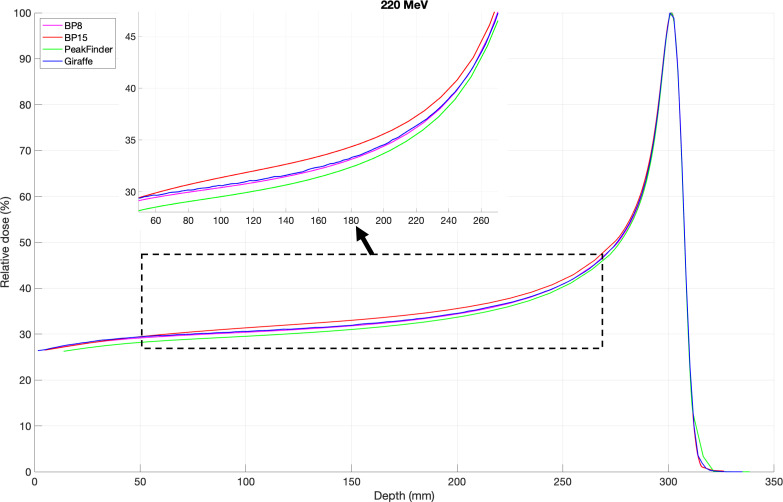
The integral depth-dose curves acquired with all chambers for 220 MeV. Abbreviations: BP8, Bragg peak chamber type 34070; BP15, Bragg peak chamber type 34089.

**Figure 1** shows a comparison of the integral depth-dose curves acquired with all chambers for 130, 150, 190, and 220 MeV. The most significant deviation between curves was located between the entrance plateau and the proximal rise of the Bragg peak, and all energy beams have the same curve arrangements. As shown in **Figure 2**, the highest curve is from PTW Bragg peak chamber type 34089 (BP15) with 15-cm diameter, followed by IBA Giraffe with 12-cm diameter, PTW Bragg peak chamber type 34070 (BP8), and PTW PeakFinder with 8-cm diameter. PTW PeakFinder has lower curves than PTW Bragg peak chamber type 34070, which could be explained by the smaller volume of water, leading to less scatter.

### The Geometric Collection Efficiency

**Figure 3.**
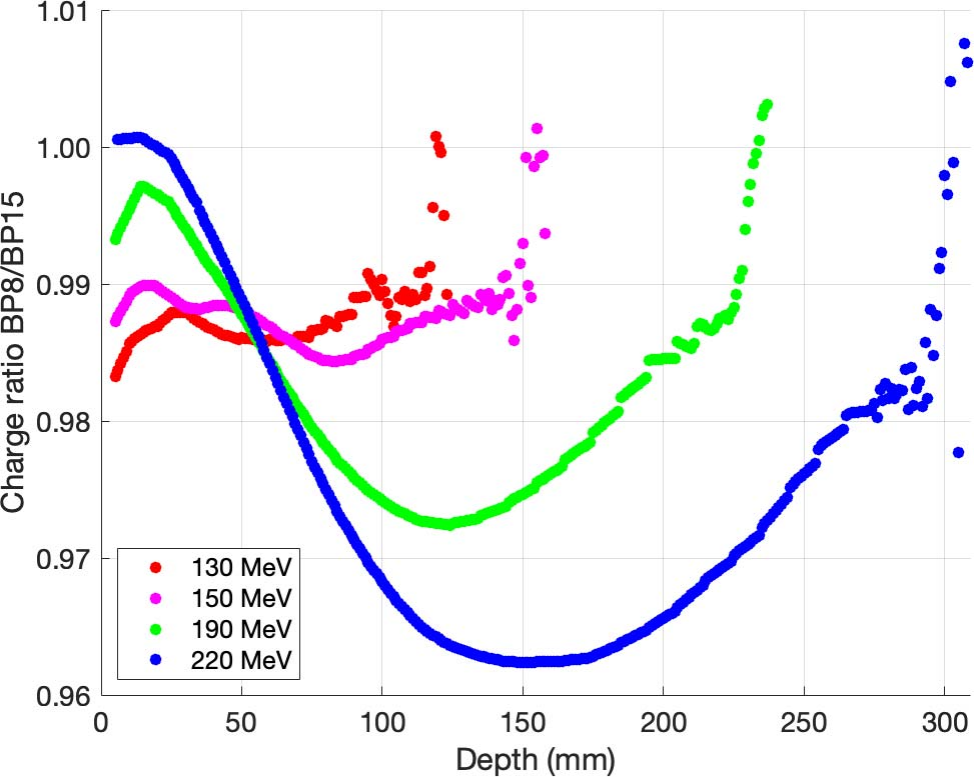
The ratios of depth-dose curves acquired with 2 types of PTW Bragg peak chamber: 34070 and 34089. Abbreviations: BP8, Bragg peak chamber type 34070; BP15, Bragg peak chamber type 34089.

**Figure 4.**
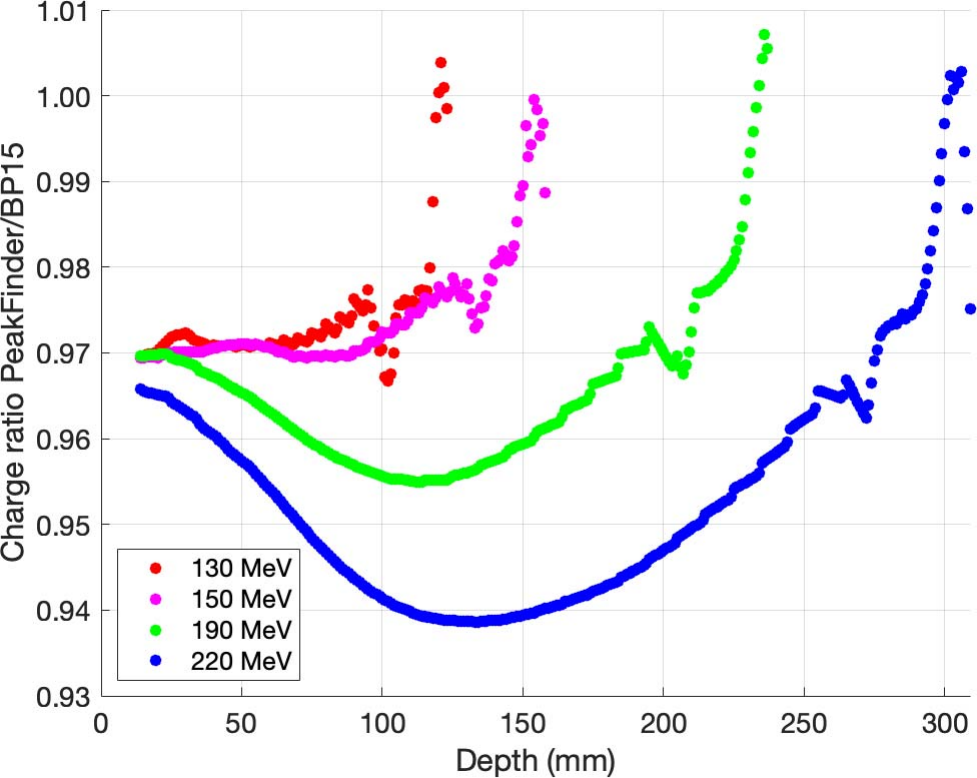
The ratios of depth-dose curves acquired with PTW PeakFinder and Bragg peak chamber 34089. Abbreviation: BP15, Bragg peak chamber type 34089.

**Figure 5.**
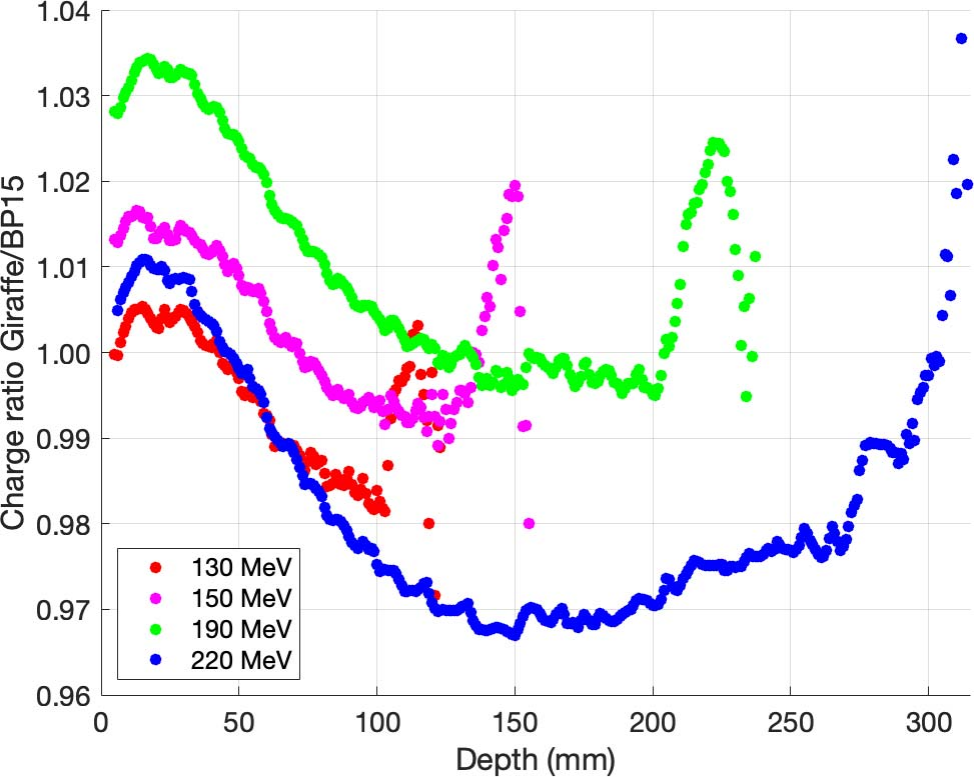
The ratios of depth-dose curves acquired with IBA Giraffe and PTW Bragg peak chamber 34089. Abbreviation: BP15, Bragg peak chamber type 34089.

All integral depth-dose curves were interpolated on a 1-mm grid and scaled in-depth to match R_80_ from NIST before calculating the ratio of each energy. The proportions of a depth-dose curve acquired with 2 types of PTW Bragg peak chambers are shown in **Figure 3**. At the intermediate depth, we found that PTW Bragg peak chamber type 34089 with the largest diameter had an increased collection efficiency, when compared to PTW Bragg peak chamber type 34070, of about 1.4%, 1.6%, 2.7%, and 3.8% for 130, 150, 190, and 220 MeV, respectively. **Figure 4** shows the ratio of a depth-dose curve acquired with PTW PeakFinder and PTW Bragg peak chamber type 34089. We found that PTW Bragg peak chamber type 34089 had an increased collection efficiency, when compared to PTW PeakFinder, of about 2.9%, 3.0%, 4.5%, and 6.1% for 130, 150, 190, and 220 MeV, respectively. **Figure 5** shows the ratio of a depth-dose curve acquired with IBA Giraffe and PTW Bragg peak chamber type 34089. We also found that PTW Bragg peak chamber type 34089 increased collection efficiency, when compared to IBA Giraffe, by about 0.8%, 0.2%, 0.1%, and 3.1% for 130, 150, 190, and 220 MeV, respectively. Furthermore, we realized that the curves acquired with IBA Giraffe vary along the depths, which could be explained by the differences in the conditions associated with IBA Giraffe compared to the other chambers, such as multi-layer ionization chamber, approximately 1.9-mm spatial resolution, and water-equivalent material.

## Discussion

PTW Bragg peak chamber type 34089 improves geometric collection efficiency by 1.4% to 3.8%, 2.9% to 6.1%, and 0.8% to 3.1% when compared to PTW Bragg peak chamber type 34070, PTW PeakFinder, and IBA Giraffe, respectively. In other words, the 15-cm detector diameter requires less correction than the PTW Bragg peak chamber type 34070, PTW PeakFinder, and IBA Giraffe. Therefore, the increasing detector diameter improves geometric collection efficiency depending on beam energy and depth, and the most significant difference was found at intermediate depths of the highest proton energy. It means that the PTW Bragg peak chamber type 34089 with the largest diameter could collect more secondary protons and reduce the inaccuracy of the halo effect, which is mainly produced by nuclear and coulomb interactions with the detection medium (eg, water); these findings agree with those of Baumer et al [4], Langner et al [2], and Mojzeszek et al [13]. Therefore, it can increase the accuracy of the proton dose model and dose calculation in the clinic. After dose modeling with the treatment planning system, verification is required, and the impact of integral depth-dose curve measurement should be investigated by using Monte Carlo methods and reported in future work. However, this study did not account for a range shifter, which is also a factor of the halo effect due to the multiple coulomb scattering. The pencil beam algorithms' dose calculation uncertainty in various treatment planning systems is insufficient to properly describe secondary protons produced in the range shifter. However, Ruangchan et al [15] discovered that 2-dose calculation algorithms of the RayStation TPS (pencil beam and Monte Carlo) have good agreement within the target in the range shifter.

The geometric collection efficiencies between the 2 detectors were close to zero at the peak region, indicating that there were fewer differences in detector sizes and halo effect, whereas higher collection efficiencies were observed at the distal fall-off region owing to detector limitations at the high-dose gradient and measurements with a step size of 5 mm. The results obtained agreed with those of Baumer et al [4] who reported a collection efficiency up to 2.0% and 3.5% between PTW Bragg peak chamber type 34070 and IBA Stingray chamber for 180 and 226.7 MeV, respectively. In addition, Mojzeszek et al [13] achieved a higher collection efficiency of 5.8% between chamber diameters of 8 and 40 cm for 226.08 MeV by using data assessed with Monte Carlo calculations. The chamber diameters that these authors used to compare with the 8-cm chamber diameter could explain these differences. For this study, we used a large-area ionization chamber with a 15-cm diameter.

## Conclusion

The integral depth-dose curves of proton pencil beams were acquired with 4 different detectors with 8-, 12-, and 15-cm diameters. The results showed that PTW Bragg peak chamber type 34089, with a 15-cm diameter, has increased geometric collection efficiency up to 3.8%, 6.1%, and 3.1% when compared to PTW Bragg peak chamber type 34070, PTW PeakFinder, and IBA Giraffe, respectively, for the highest energy. We conclude that a larger plane-parallel ionization chamber could increase the geometric collection efficiency of the detector, especially at intermediate depths and high-energy proton beams, with less difference in the Bragg peak region. PTW PeakFinder and IBA Giraffe have limitations in the measurement; therefore, they should be used mainly for fast quality assurance and should not be introduced into a treatment planning system as the input data.
